# A Practical Treatise on Uterine Hemorrhage, in Connexion with Pregnancy and Parturition

**Published:** 1832-07

**Authors:** 


### BIBLIOGRAPHICAL NOTICE.

*A Practical Treatise on Uterine Hemorrhage, in connexion with Pregnancy and Parturition.* By John T. Ingleby, Member of the Royal College of Surgeons in London, one of the Surgeons to the General Dispensary, Surgeon to the Magdalen Asylum, and Lecturer on Midwifery at the School of Medicine in Birmingham.—8vo. pp. 276. Longman, London.

It is often asserted, and it is perhaps generally believed, that he who clearly comprehends a subject can clearly explain that subject to others. We believe there are many exceptions to this axiom, and we are willing to hope that in Mr. Ingleby we have one example; and that, although his style of writing is diffuse to the last degree, and his meaning not unfrequently obscured by a barren abundance of words, he may still be a good practitioner at the bed-side of the patient. We admit that there is room for a good practical treatise on Uterine Hemorrhage; and, when we opened the present volume, we were in hopes that we should have the gratification of applauding Mr. Ingleby for having supplied the deficiency, and of recommending his book to those for whose use and instruction it is chiefly intended, namely, students and junior practitioners of midwifery. We cannot conscientiously do either the one or the other; and, with every disposition to err rather on the side of bestowing too much than too little praise, we do not feel ourselves justified in applying to the present production even the cold term of approbation, "respectable." As, in our judgment, then, Mr. Ingleby has failed in his attempt to give to obstetrical students a concise and satisfactory work on the very important subject of uterine hemorrhage, we must advise them to turn for information to the essays of RIGBY and BURNS, and the chapters by MERRIMAN and DEWEES, in their respective works. The latter especially has treated this subject in a very clear and practical manner, in his "System of Midwifery." The perusal of the hundred pages which he has dedicated to it, will much better repay the reader than the ill-arranged and ill-digested matter which occupies the 276 pages of the volume which we are compelled thus briefly to dismiss. The pages are thickly printed with "words that bear the semblance of worth, not substance."

